# Immunohistochemical expression of the glucose transporters Glut-1 and Glut-3 in human malignant melanomas and benign melanocytic lesions

**DOI:** 10.1186/1756-9966-27-34

**Published:** 2008-09-02

**Authors:** Paola Parente, Antonella Coli, Guido Massi, Antonella Mangoni, Manuela M Fabrizi, Giulio Bigotti

**Affiliations:** 1 Department of Anatomic Pathology, Catholic University "Sacro Cuore", Rome, Italy

## Abstract

**Background:**

Reported data indicate that cancer cells have increased rates of glucose metabolism, as determined by 18FDG-PET imaging in patients with malignancies. The results of many studies have demonstrated that the expression of glucose transporters, especially Glut-1, is increased in a variety of malignancies. This study was undertaken to assess the differential expression of Glut-1 and Glut-3 by benign and malignant melanocytic lesions.

**Methods:**

Immunohistochemical staining for Glut-1 and Glut-3 was performed on paraffin-embedded tissue sections prepared from melanocytic nevi (12 cases), Spitz nevi (12 cases) and primary cutaneous malignant melanomas (20 cases).

**Results:**

We observed immunoreactivity for Glut-1 in all melanocytic nevi, 9 of the 12 Spitz nevi and in 9 of the 20 malignant melanomas, whereas Glut-3 was expressed in all the melanocytic lesions, both benign and malignant.

**Conclusion:**

These findings indicate that the glucose transporters Glut-1 and Glut-3 play a role in the glucose metabolism of melanocytic cells. Glut-1 was present in the majority of benign nevi, whereas its expression was downregulated in 55% of malignant melanomas. Our results suggest that glucose transporter Glut-1 expression can significantly discriminate between human malignant melanoma and benign melanocytic nevi, and support the idea that additional mechanisms other than Glut-1 may contribute to glucose uptake in melanomas.

## Background

Glucose transporters (Glut-1-14) belong to a family of structurally-related proteins that mediate energy-independent glucose transport across the plasma membrane. These transporters differ in their tissue distribution and affinity for glucose [1,2]. Glut-1 was the first member of the facilitated glucose transporter family identified [1]. It is mostly expressed in erythrocytes, endothelial cells of the blood-brain barrier and placental cells [3]. The human Glut-1 gene, which has been localized to the short arm of chromosome 1 (1p34.2), is 35 kb in length and contains 10 exons that encode a protein of 492 aminoacids. This protein is highly conserved among different species that include human, rat, mouse and pig [4].

There is evidence that human malignancies, in which glucose metabolism is increased, express higher levels of Glut-1 than do normal cells. In particular, human Glut-1 is overexpressed in malignant cells and in a variety of tumours, that include the breast, pancreas, cervix, endometrium, lung, mesothelium, colon, bladder, thyroid, bone, soft tissues, and oral cavity [3,5-14]. Specifically, Glut-1 expression has been associated with increased malignant potential, invasiveness, and a poor prognosis [6,9,14,15]. Glut-3 is also expressed in human malignant tissue, but there are discrepancies among the reported results [9,16,17]. To the best of our knowledge, immunoreactivity of Glut-1 has not previously been shown for melanocytic lesions, and immunoreactivity for Glut-3 has not been investigated on these lesions [18]. We used immunohistochemistry to evaluate the expression of Glut-1 and Glut-3 in human benign nevi and malignant melanomas.

## Methods

### Tissue samples

We selected, retrospectively, 44 specimens from patients who had undergone surgery for either benign or malignant melanocytic lesions. The project was approved by the Committee of Ethics at the Catholic University "Sacro Cuore", Faculty of Medicine, Rome, Italy. Cases were retrieved from the archives of the Pathology Laboratory, Columbus Clinic, Catholic University "Sacro Cuore" (Rome, Italy). The histological diagnoses were as follows: melanocytic nevus, n = 12; Spitz nevus, n = 12; and primary cutaneous malignant melanoma, n = 20. The patients with melanomas were subdivided into two groups: the first contained ten patients whose tumour thickness was less than or equal to 1 mm, whereas the second contained ten patients whose tumour thickness was more than 1 mm. All diagnoses had been made on the basis of histopathological features that were evident in sections routinely stained with haematoxylin and eosin. The criterion for lesion selection was the almost absence or paucity of melanin to allow a good immunohistochemical evaluation. Table 1 summarizes the clinical and pathological characteristics of the patients who were affected by malignant melanomas. A sentinel lymph node biopsy was performed on all patients with melanoma.

**Table 1:** Clinical and pathological findings of the patients affected by malignant melanoma

**Patient n.**	**Age**	**Sex**	**Site**	**Thickness (mm)**	**S-LFN**	**LFN**
*Thickness ≤ 1 mm*						
1.	58	F	Groin	0.7	-	
2.	34	F	Thigh	0.3	-	-
3.	41	M	Underscapular	0.88	-	
4.	32	M	Costal	0.4	-	
5.	50	M	Upperscapular	0.3	-	+
6.	34	F	Back	0.5	+ (itc)	-
7.	66	M	Lumbar	0.5	+ (itc)	-
8.	64	M	Paravertebral	0.5	-	
9.	63	F	Leg	1	-	
10.	70	M	Thorax	0.5	-	
*Thickness > 1 mm*						
1.	36	F	Thigh	1.2	-	
2.	55	F	Knee	1.2	-	
3.	31	M	Back	1.4	+ (itc)	-
4.	63	F	Forearm	1.1	-	
5.	35	F	Thigh	2	+	-
6.	30	F	Thigh	1.7	-	
7.	33	M	Arm	1.4	-	
8.	62	F	Arm	3	-	
9.	38	F	Pubis	2.7	+	-
10.	59	F	Leg	2	-	

S-LFN, sentinel lymph node; LFN, regional lymphadenectomy; itc, isolated tumour cells

### Immunohistochemistry

Immunohistochemical analysis was performed on 4 μm paraffin sections of the tumours using an automatic immunostainer (BenchMark; Ventana Medical Systems, Tucson, AZ, USA). The sections were incubated for 24 min at 37°C with a rabbit polyclonal antibody against Glut-1 (1:200; Diapath, Bergamo, Italy) and a rabbit monoclonal antibody against Glut-3 (1:1000; Diapath). The immunostaining was visualized using Xt Ultra View Red (Diapath) as a chromogen, according to the manufacturer's instructions. Cell nuclei were lightly counterstained with standard Mayer's haematoxylin. Erythrocytes and keratinocytes served as internal positive controls for Glut-1. Samples of placenta and from gray matter of the cerebral hemispheres were used as positive controls for Glut-3. For negative controls, the primary antibodies were omitted.

Sections that had been immunostained for Glut-1 or Glut-3 were independently assessed by two observers (PP and GM). Where the results were ambiguous, an agreement was reached after discussion. The results of the immunohistochemical analysis were evaluated according to the intensity of the staining as follows: 0, absent or barely-detectable staining; 1, weak staining; 2, moderate staining; and 3, strong staining. The Glut-1 staining intensity was considered strong when it was equal to that in red blood cells in the same sections.

### Statistical analysis

Two-tailed Fisher's exact test was used to analyze the contingency tables and *P *< 0.05 was considered to be statistically significant. Statistical analyses were performed by JMP software (SAS Institute Inc., CARY, NC, USA).

## Results

The normal keratinizing epithelium in samples from all the patients showed immunoreactivity for Glut-1 and Glut-3 in the basal cell and malpighian layers: the signal for Glut-1 was strong, whereas that for Glut-3 appeared weak to moderate. The immunoreactivity of Glut-1 was always strongly intense in erythrocytes, eccrine sweat glands and the perineurium of small nerve trunks. In contrast, the antibody against Glut-3 stained the vascular endothelium together with a number of dendritic cells throughout the squamous cell layer. The immunohistochemical results for all the melanocytic lesions are summarized in Table 2. In all the cases studied, staining of Glut-1 and Glut-3 occurred in both the plasma membrane and the cytoplasm of the tumour cells. Within the cytoplasm, the staining pattern was granular.

**Table 2:** Immunohistochemical expression of Glut-1 and Glut-3 in benign melanocytic lesions and malignant melanomas

**Histological diagnosis**	**Glut-1 expression (%)**				**Glut-3 expression (%)**			
	**0**	**1**	**2**	**3**	**0**	**1**	**2**	**3**
Benign melanocytic lesions (*n = 24*)	3(13)	13(54)	7(29)	1(4)	0	3(1)	5(21)	16(67)
Malignant melanoma (*n = 20*)	11(55)	8(40)	1(5)	0	0	3(15)	1(5)	16(80)

### Benign lesions

**Figure 1:**
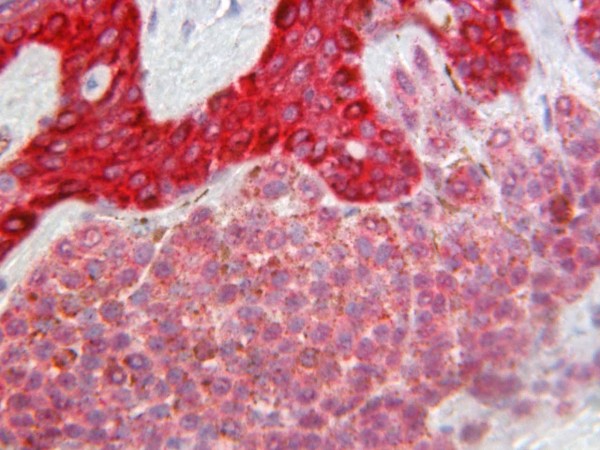
Melanocytic nevus with moderate staining for Glut-1.

**Figure 2:**
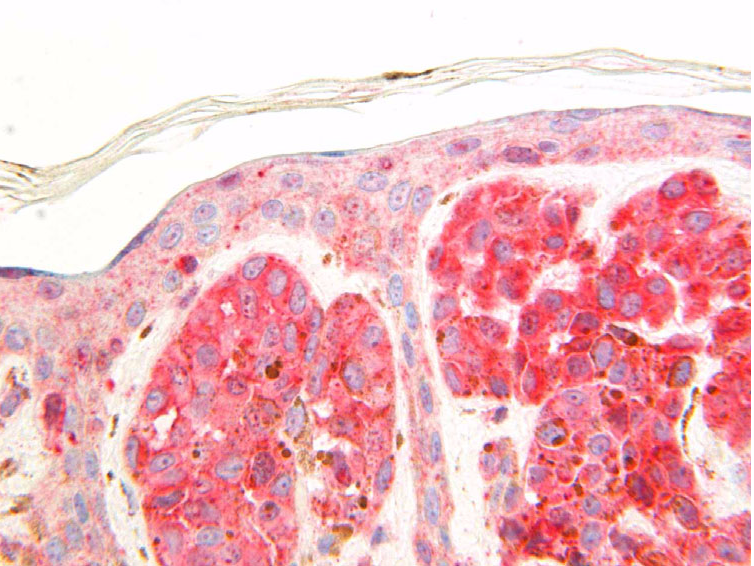
Strong immunoreactivity for Glut-3 in a melanocytic nevus.

All the melanocytic nevi (12) were positive for both Glut-1 and Glut-3. Glut-1 immunoreactivity was weak in five cases, moderate in six cases [Figure 1], and intense in one case. The immunoreactivity for Glut-3 was weak in one case, moderate in five cases, and intense in six cases [Figure 2].

**Figure 3:**
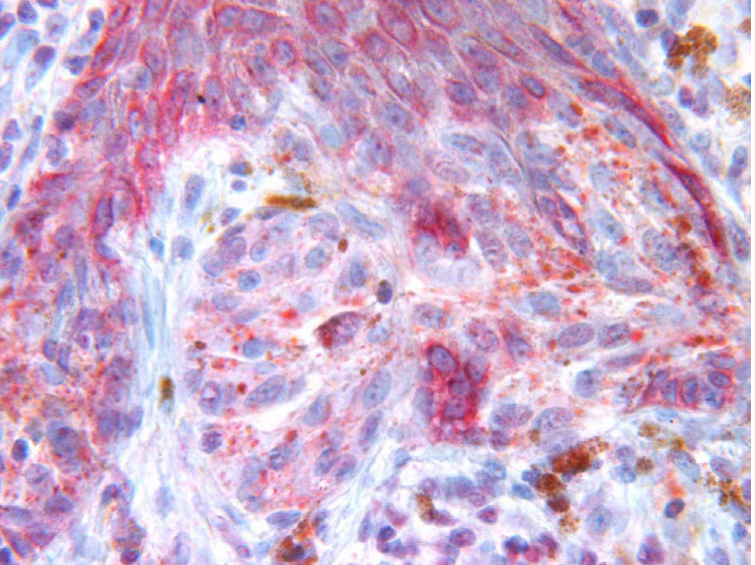
Spitz nevus showing weak immunoreactivity for Glut-1.

Among the twelve cases of Spitz nevus, positive immunostaining for Glut-1 was observed in nine cases. Eight of these showed weak immunoreactivity [Figure 3], and one showed moderate immunoreactivity. Only three cases of Spitz nevi did not show Glut-1 immunoreactivity. Glut-3 expression was evident in all cases, and ten showed strong expression. Only two cases were weakly positive. Therefore, nine cases were positive for both Glut-1 and Glut-3.

Overall, among the 24 benign melanocytic lesions, Glut-1 was expressed in 21 cases (87.5%), and Glut-3 in all cases (100%).

### Malignant melanomas

Of the ten tumours in the group with thickness ≤ 1 mm, five were positive for the expression of Glut-1, and immunoreactivity was not or barely detected in the remaining five specimens. All ten tumours were positive for Glut-3. The intensity of staining for Glut-3 was weak in two cases, moderate in one case, and intense in seven cases. Therefore, five cases showed the concurrent expression of Glut-1 and Glut-3.

**Figure 4:**
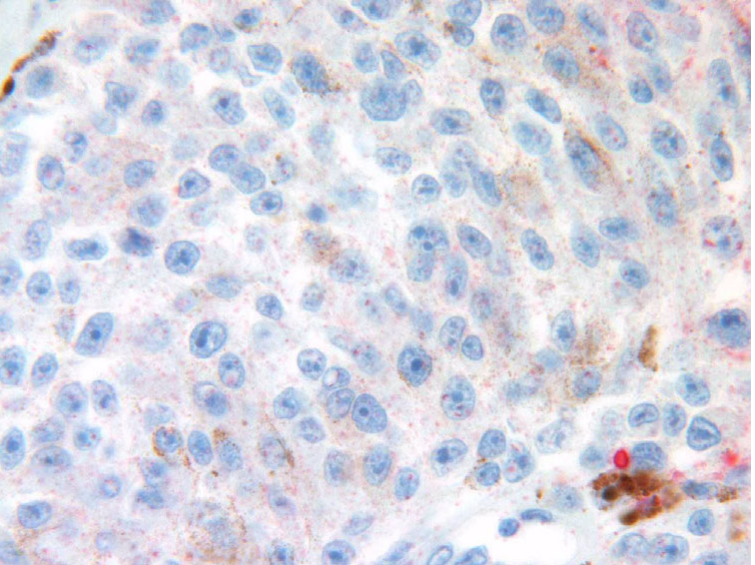
Glut-1 is not expressed in a malignant melanoma > 1 mm thick.

**Figure 5:**
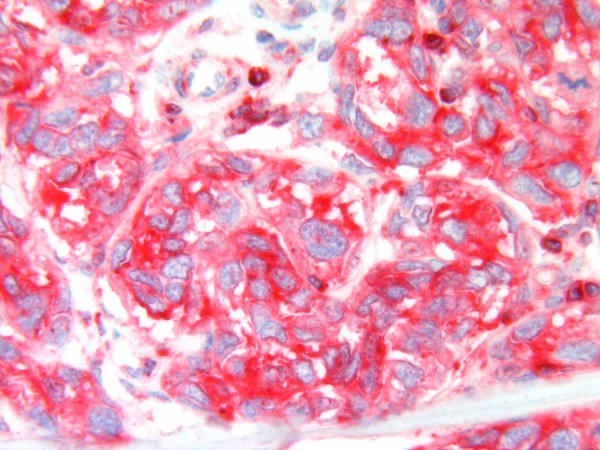
Strong immunoreactivity for Glut-3 in a malignant melanoma > 1 mm thick.

Of the ten cases in the group with thickness > 1 mm, Glut-1 immunoreactivity was present in four tumours, with weak immunoreactivity in three cases and moderate immunoreactivity in one case. Six tumours did not show Glut-1 immunoreactivity [Figure 4]. Glut-3 expression was evident in all tumours, with intense immunoreactivity in nine cases [Figure 5] and weak immunoreactivity in one case. Four cases were positive for both Glut-1 and Glut-3.

### Statistical analysis

For statistical purposes, the 44 specimens were categorized in two groups: benign melanocytic lesions, which comprised melanocytic nevi and Spitz nevi, and malignant melanomas. The results of the statistical analysis showed that Glut-1 was significantly expressed in benign lesions as compared to malignant melanomas (p = 0.0118). Conversely, Glut-3 was expressed in all benign and malignant melanocytic lesions, with no significant differences. With regard to the malignant melanomas, no significant correlation was found between Glut-1 expression and the thickness of the tumour.

## Discussion

Reported data indicate that glucose transporters, in particular Glut-1, play an important role, although not completely yet understood, in tumour progression. Glut-1, whose expression can be induced in cancer cells by oncogenes, growth factors, interleukin-1, local hypoxia and inflammatory changes, enhances the activity of the matrix metalloproteinases, whose activities have been directly related to tumour invasiveness and metastasis [2,6,9,19]. Several reports indicate that the expression of the glucose transporters Glut-1 and/or Glut-3 is upregulated in many neoplasms and correlates with the uptake of the glucose analogue 18FDG in a variety of human tumours [20-24].

To the best of our knowledge, there have not been any previous immunohistochemical studies that have successfully detected Glut-1 and Glut-3 in melanocytic lesions. In our study, immunoreactivity of Glut-1 was detected in 100% of the melanocytic nevi, in 75% of the Spitz nevi and in 45% of the melanomas, whereas Glut-3 was expressed in all the melanocytic lesions, both benign and malignant. The proportion of the benign tumours which expressed Glut-1 was significantly higher than that of the malignant tumours which were positive for Glut-1. We also observed that the intensity of immunoreactivity for Glut-1 was higher in benign lesions than in malignant ones. These findings suggest that Glut-1 and Glut-3 play a role in the regulation of glucose metabolism in human melanocytic lesions.

Although Breslow's thickness remains the most important prognostic factor for cutaneous melanoma, our study has shown no significant correlation between Glut-1 expression and the thickness of the tumour [25]. Additional studies seem to be necessary to confirm the data.

The variation in the immunoreactivity that we obtained in melanomas is in agreement with the results of the study by Wachsberger et al, in which a wide variability in Glut-1 levels as detected by western immunoblotting on human melanomas was reported [26]. Also, our findings agree with a number of reports that indicate that metabolic imaging with 18FDG is not a sensitive indicator of occult metastases in patients with malignant melanoma [27-30].

The results of our study have significant differences when compared with those of previous studies, which demonstrated that Glut-1 is overexpressed in human tumours. Firstly, most of the published reports studied epithelial neoplasms, whereas nevi and malignant melanomas arise from melanocytes, which originate from the neural crest. It is well known that melanocytes and derived cells express antigens that are not found in epithelial elements. Secondly, the tumours previously described in the medical literature showed variable intratumoural expression of Glut-1 with an intense immunoreactivity in aggressive regions of the tumour, such as the poorly differentiated and central hypoxic areas [9]. However, we observed homogeneous Glut-1 expression throughout the tumours examined. In our study, all positive samples (benign and malignant) showed a membranous and cytoplasmic immunoreactivity for both Glut-1 and Glut-3, independent of staining intensity, whereas other researchers have demonstrated a cytoplasmic and/or membranous staining pattern [7,11,21]. It should be noted that an increase in the immunoreactivity of Glut-1 and the membranous staining pattern have been reported as indicators of hypoxia [11,31-34]. Finally, the melanomas were much smaller in volume and had less cellularity than the carcinomas, and did not contain any cystic, necrotic or haemorrhagic components.

In the medical literature, we have found only one study that investigated Glut-1 in nevi and melanomas, and the authors reported that all the melanocytic lesions examined were negative for Glut-1 expression [18]. These differing results could be due to the use of a different antibody. Nevertheless, we are unaware of any previous study that examined Glut-3 expression in benign and/or malignant melanocytic lesions.

## Conclusion

Our study clearly shows for the first time that Glut-1 is expressed in all melanocytic nevi and the majority of Spitz nevi, whereas Glut-1 expression is downregulated in 55% of malignant melanomas. However, Glut-3 is present in malignant melanomas as well as in benign lesions. Our results suggest that human malignant melanomas and benign nevi show a differential expression of the glucose transporter Glut-1. Also, these data support the idea that, in a number of melanomas, other key factors, i.e. other glucose transporter isoforms, rather Glut-1, or other processes may contribute to glucose transport across the cell membrane. However, additional studies needed to further explore glucose metabolism of melanocytic lesions.

## Competing interests

The authors declare that they have no competing interests.

## Authors' contributions

PP conceived of the study, collected the data and drafted the manuscript. AC designed this study and modified the manuscript. GM procured reagents, supervised the experimental work and performed microphotography. AM and MMF carried out the immunohistochemical analyses. GB participated in the designed of the study, and has been involved in revising the manuscript. All authors read and approved the final manuscript.
